# Bayesian Analysis of AI-Driven Cost Savings in UK and Australian Health Care Systems: Cross-Sector Implementation Study

**DOI:** 10.2196/89113

**Published:** 2026-08-26

**Authors:** Jayanta Sarkar, Christopher Drovandi, Sandeep Reddy

**Affiliations:** 1 School of Economics and Finance Queensland University of Technology Brisbane, Queensland Australia; 2 Queensland University of Technology Brisbane, Queensland Australia; 3 Queensland University of Technology Kelvin Grove, Queensland Australia

**Keywords:** AI, artificial intelligence, Bayesian analysis, cost savings, health care economics, Monte Carlo simulation, sequential Monte Carlo

## Abstract

**Background:**

Health care systems face growing fiscal pressure while AI reaches clinical parity in several domains. UK National Health Service expenditure rose by 52%, while Australia's health expenditure grew by 29% between 2019 and 2023. Yet large-scale cost savings from AI remain limited, largely because implementation constraints continue to outweigh technical capability.

**Objective:**

This study develops a Bayesian budget-impact framework to estimate AI-driven gross cost savings in radiology, workflow optimization, and workforce optimization in the United Kingdom and Australia, explicitly accounting for adoption uncertainty, effectiveness, and implementation risk.

**Methods:**

We used a sequential Monte Carlo simulation with 1000 particles to estimate annual gross cost savings. The core savings function combined expenditure base, sector weight, adoption, effectiveness, and implementation risk. Priors were informed by a structured review of multiple studies per domain. The savings likelihood used a heteroscedastic noise specification in which the SD followed an exponential prior, with the mean set to 15% of each sector’s observed savings estimate, ranging from US $12.0 million for Australian radiology to US $87.2 million for UK workforce optimization. The likelihood was also augmented with sector-specific observations that anchored effectiveness to published cost-reduction estimates and implementation risk to observed deployment failure rates. Scenario analysis applied multipliers to 1000 bootstrap posterior draws across optimistic, conservative, and pessimistic settings. Sensitivity analyses varied the *σ* scaling factor from 0.10 to 0.20 and perturbed prior means for adoption, effectiveness, and implementation risk by ±20%.

**Results:**

Posterior annual savings were US $949 million (95% credible interval [CrI] US $720.6-US $1173.5 million) for the United Kingdom and US $737 million (95% CrI US $526.1-US $953.6 million) for Australia. Workforce optimization generated the largest share of savings in both countries, contributing 62.4% of UK savings (US $591.9 million, 95% CrI US $398.8-US $854.8 million) and 76.2% of Australian savings (US $561.4 million, 95% CrI US $331.9-US $815.3 million). Posterior implementation risk estimates ranged from 35.5% to 47.4%, below prior means of 49.1% to 57.2%, reflecting the empirical anchoring introduced through deployment-failure data. Across scenarios, projected savings ranged from US $357 million to US $1.845 billion in the UK and from US $267.2 million to US $1.454 billion in Australia. Baseline cumulative projections for 2024-2030 were US $10.1 billion for the United Kingdom and US $8.0 billion for Australia. The sensitivity analyses confirmed the robustness of the posterior savings estimates.

**Conclusions:**

AI could generate substantial expenditure reductions in both health systems, but implementation risk remains the main constraint on realizing those gains. Workforce savings should be interpreted primarily as capacity gains that can be redeployed to higher-value care, not as automatic cash savings. The Bayesian framework offers probabilistic planning ranges rather than point forecasts and provides a practical basis for policy planning under uncertainty.

## Introduction

Health care systems worldwide face an unusual convergence of fiscal pressure and technological opportunity. In the United Kingdom, National Health Service expenditure rose from £192.8 billion in 2019 to £292 billion in 2023, a 51% increase that substantially outpaced both inflation and economic growth [1]. In Australia, health care expenditure increased by 29% to Aus $252.5 billion over the same period, with hospital costs alone accounting for 42.4% of total health spending [2]. At the same time, AI has demonstrated clinical-grade performance across a wide range of health care applications, from diagnostic imaging to administrative workflows [3-6]. Yet despite growing AI adoption, large-scale, measurable cost savings remain elusive [7-9].

This gap between AI’s technological maturity and its limited economic impact appears to stem less from algorithmic limitations than from implementation frameworks that fail to accommodate the probabilistic nature of AI effectiveness and the shifting risk profile of health care technology adoption. Traditional health economic models perform well for pharmaceuticals and medical devices, but they are less well suited to AI implementations, where efficacy is not static and scalability is rarely linear. Evidence suggests that roughly 50% to 57% of health care AI deployments fail to achieve their intended outcomes, defined here as failing to demonstrate the projected cost savings or clinical improvements within the planned implementation horizon rather than merely failing to deploy technically [8,10]. That pattern points to implementation, rather than technical capability alone, as the primary bottleneck.

The existing literature reveals a striking disconnect between theoretical promise and documented savings. Vendors and policy narratives often emphasize AI’s cost-saving potential, but peer-reviewed evidence continues to show how difficult it is to realize those gains at scale [10]. This disconnect reflects both implementation challenges and weaknesses in existing economic evaluation methods. Recent reviews point to 3 main approaches to health care AI economic evaluation, each with clear limitations.

Performance analyses, exemplified by the evaluation of chest x-ray AI by Rajpurkar et al [11], provide point estimates but fail to capture uncertainty in both AI performance and implementation contexts. Markov and decision-theoretic models can represent temporal dynamics, but they depend on fixed transition probabilities, an assumption that fits poorly with the learning effects and adoption variability typical of AI deployment [12]. Monte Carlo simulations address some dimensions of uncertainty, yet conventional frequentist formulations are less effective at incorporating prior knowledge drawn from heterogeneous implementation studies [13].

Bayesian methods offer a more natural framework for this problem. In health technology assessment, Spiegelhalter et al [14] showed how Bayesian hierarchical models can synthesize evidence across heterogeneous clinical settings while preserving uncertainty. In health care AI, Chen et al [15] used Bayesian methods to evaluate diagnostic performance across multiple hospitals and found substantial between-institution variability that conventional approaches had missed. However, most existing work of this kind has focused on clinical effectiveness rather than economic outcomes, leaving a clear methodological gap in the evaluation of AI-driven cost savings.

Current health care AI economic evaluations suffer from several fundamental limitations. First, deterministic models do not adequately capture uncertainty in either AI performance or the implementation context. Fixed effectiveness assumptions, for example, overlook the substantial variation reported across health care settings, where identical AI systems may achieve effectiveness estimates ranging from 15% to 45%, depending on institutional conditions [16]. Second, many models understate implementation risk, even though recent evidence identifies it as the main constraint on realized AI savings. Studies of deployed health care AI systems found that implementaion barriers, rather than technical limitations, are the primary obstacles to achieving projected savings [17]. Third, many evaluations rely on short-run clinical trial evidence that does not reflect learning effects, gradual adoption, or workflow adaptation over time [18,19]. Fourth, few studies provide probabilistic savings estimates that policymakers can use directly for planning.

We address those gaps with 2 questions. First, what are the projected economic impacts of AI implementation across radiology, workflow optimization, and workforce optimization in the UK and Australian health care systems once uncertainty in effectiveness, adoption, and implementation risk is taken into account? Second, how do implementation risks affect the realization of projected AI cost savings, and how do those risks vary across sectors and national settings?

## Methods

### Study Design

We conducted a Bayesian budget-impact simulation to estimate AI-driven cost savings across 3 health care sectors in the United Kingdom and Australia. Rather than fitting a traditional hierarchical model, we used a Bayesian simulation framework that synthesizes evidence from heterogeneous implementation studies while explicitly incorporating uncertainty in effectiveness, adoption, and implementation risk. The primary time horizon was 2024 for the cross-sectional analysis and was extended to 2024-2030 using inflation-adjusted temporal projections based on annual inflation rates of 4% for the United Kingdom and 5% for Australia.

We adopted a payer/provider perspective, using the National Health Service (NHS) for the United Kingdom and the Australian Government for Australia. The primary estimand was annual gross cost savings, defined as the reduction in expenditure attributable to AI-enabled efficiency gains rather than cost offsets or net savings after implementation costs. We therefore did not model implementation costs such as software licensing, system integration, staff training, governance, or cybersecurity. Nor did we model health outcomes, quality-adjusted life-years, or incremental cost-effectiveness ratios because our objective was a budget-impact analysis rather than a cost-effectiveness evaluation.

### Data Sources and Sector Specifications

We drew health care expenditure data from official national statistics for the 2022-2023 period. For the United Kingdom, we used the Office for National Statistics (ONS) estimate of £292 billion in total health care expenditure [1]. For Australia, we used the Australian Institute of Health and Welfare estimate of Aus $252.5 billion [2]. We converted all monetary values to US dollars using International Monetary Fund (IMF) December 2023 spot exchange rates of £1=US $1.27 and the Reserve Bank of Australia (RBA) December 2023 spot rate of Aus $1=US $0.68. We used spot rates because they align with the official expenditure reports. Purchasing power parity rates could alternatively be used for welfare comparisons, but they would not materially alter either the direction or the rank ordering of our results. Full expenditure-base calculations are reported in Table S3 in Multimedia Appendix 1.

We derived sector expenditure weights from ONS, the Australian Institute of Health and Welfare (AIHW), and OECD: Organization for Economic Co-operation and Development (OECD) health accounts [1,2,20,21]. Radiology accounted for 0.83% of total health care spending in the United Kingdom and 2.8% in Australia [22-24]. Workflow optimization, defined here as health administration and clinical workflow process costs, accounted for 2% of UK expenditure (approximately £5.84 billion) and 2.77% of Australian expenditure (approximately Aus $7.0 billion) [1,2]. Workforce optimization, capturing compensation of employees, accounted for 30% of UK health care expenditure and, for Australia, was parameterized using the workforce payroll component of recurrent public hospital expenditure, equal to Aus $56.62 billion (representing 52.9% of recurrent public hospital expenditure and an effective model weight of 22.4% of total Australian health care expenditure) [20,21]. These shares imply approximate sector bases of £2.4 billion (US $3.05 billion) for UK radiology, Aus $7 billion (US $4.76 billion) for Australian radiology, £5.84 billion (US $7.42 billion) for UK workflow optimization, Aus $7.0 billion (US $4.76 billion) for Australian workflow optimization, £87.6 billion (US $111.25 billion) for UK workforce optimization, and Aus $56.62 billion (US $38.5 billion) for Australian workforce optimization [1,2,20-24].

As detailed below, our structured review yielded only 3 to 4 studies per domain, although all had sample sizes of at least 100 participants. That relatively sparse evidence base motivated the use of informative priors and underscores the need for future meta-analyses and registry-based evidence.

### Bayesian Model Structure and Sampling

We modeled annual cost savings (*S*) for sector *i* in country *j* as:



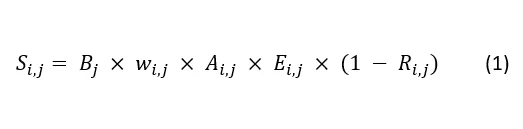



where *B_j_* represents base health care expenditure, *w_i,j_* denotes sector weight, *A_i,j_* is the adoption rate, *E_i,j_* represents AI effectiveness, and *R_i,j_* captures implementation risk. In practical terms, equation 1 multiplies total health care spending by the sector’s share, the fraction of activity that adopts AI, the cost-reducing effect of AI, and the probability that implementation succeeds, yielding each sector’s expected cost saving. The multiplicative specification treats adoption rate *A* and implementation risk *R* as statistically independent. Although higher-risk environments may exhibit slower AI adoption in practice (introducing negative correlation), the current literature does not provide joint (*A*, *R*) data sufficient to parameterize a bivariate distribution. The independence simplification is therefore a necessary modeling choice whose conservative implications—a modest upward bias in projected savings—are discussed in the Limitations section.

We assumed that observed cost savings followed a normal distribution centered on the value implied by equation 1. We treated the SD, *σ*, as an estimable parameter and assigned it a sector-country-specific exponential prior with a mean equal to 15% of the observed savings estimate for that sector-country pair. This heteroscedastic specification scales absolute estimation noise to the magnitude of projected savings, so sectors with larger projected savings also carry larger absolute uncertainty. The implied prior mean values for ranged from US $12.0 million for Australian radiology to US $87.2 million for UK workforce optimization, reflecting the variation in observed savings across sectors.

The savings equation links 3 sector-specific latent parameters—adoption rate (*A*), effectiveness (*E*), and implementation risk (*R*)—to 1 or more constructed savings observations per sector-country pair, derived from the eligible literature and converted to comparable annual monetary values. In the current implementation, radiology uses 2 constructed savings anchors per country, whereas workflow optimization and workforce optimization each use 1. To improve identification, we augmented the savings likelihood with sector-specific auxiliary observations for effectiveness and implementation failure so that the joint log-likelihood is the sum of the savings term, the effectiveness-observation term, and the implementation-failure term. The augmented log-likelihood is given by equation 2:



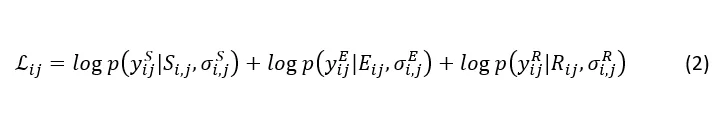



where *y^S^_ij_*, *y^E^_ij_*, and *y^R^_ij_* denote the observed savings, effectiveness, and implementation-failure anchors, respectively, with corresponding measurement uncertainties *σ^S^_i,j_*, *σ^E^_i,j_*, and *σ^R^_i,j_* derived from the literature. This specification anchors *E* to literature-based cost-reduction estimates and *R* to empirical deployment-failure rates, while adoption *A* is estimated conditional on those anchored quantities. The corresponding observed inputs and measurement uncertainties are described in the Prior Distributions section, and Table S1 in Multimedia Appendix 2 reports the observed cost-savings values used for the United Kingdom and Australia. We note that with 3 latent parameters (*A*, *E*, *R*) per sector-country pair—updated against 2 constructed savings observations for radiology and 1 each for workflow and workforce optimization—the model is formally underidentified. Equation 2 partially addresses this by anchoring *E* and *R* with independent observations, but residual sensitivity to prior specifications remains and cannot be fully eliminated without sector-specific multioutcome datasets. A prior sensitivity analysis reported in Multimedia Appendix 3 demonstrates that sector rank orderings and order-of-magnitude savings estimates are robust to ±20% perturbations in individual prior means and to alternative *σ* scaling factors.

We estimated posterior distributions using sequential Monte Carlo (SMC) with 1000 particles [25,26]. We chose SMC over conventional Markov chain Monte Carlo (MCMC) because it requires relatively little tuning and is well suited to analyzing multiple datasets. The SMC algorithm samples through a sequence of intermediate distributions that gradually transform the prior into the posterior by annealing the likelihood. At each iteration, we selected the next temperature so that the effective sample size remained close to 500. We then resampled to eliminate low-weight particles and replicate high-weight particles. After resampling, we applied an MCMC kernel to perturb duplicated particles and reduce particle degeneracy. The number of MCMC iterations was set adaptively using the estimated acceptance rate. Throughout the algorithm, the number of unique particles remained above 900, indicating stable sampler performance. This process is described in Table S3 in Multimedia Appendix 2.

### Prior Distributions

We constructed prior distributions from a structured review of major health care AI studies with sample sizes of at least 100 participants and quantifiable effectiveness estimates. Complete parameter values are reported in Table S1 in Multimedia Appendix 1, and Table S2 in Multimedia Appendix 1 maps those values to the underlying literature.

The effectiveness parameter *E_i,j_* is defined domain-specifically and is not directly commensurate across sectors. In radiology, *E* represents the proportional reduction in reading cost per screening episode attributable to AI triage. In workflow optimization, *E* represents the proportional reduction in cost per treatment course or workflow episode. In workforce optimization, *E* represents the proportional reduction in overtime and excess scheduling costs. These cost categories are mutually exclusive by design: radiology savings affect reading cost per episode; workflow savings affect process cost per course; workforce savings affect labor allocation costs. Adoption rate (*A*) represents the proportion of eligible activities within the sector that are subject to AI-assisted delivery. Implementation risk (*R*) represents the probability that a given AI deployment fails to achieve its projected savings within the planned implementation horizon.

For effectiveness, we used truncated normal (TN) priors that reflect both the empirical literature and domain-specific constraints. In radiology, studies by Lång et al [27] and Marinovich et al [28] show that AI-assisted screening can improve diagnostic accuracy and workflow efficiency, although formal modeling of cost-effectiveness or combined deployment impact remains limited [29]. We therefore specified UK radiology effectiveness as TN(0.459, 0.128; lower=0.01, upper=0.6). Workflow optimization used TN(0.55, 0.12; lower=0.01, upper=0.8), anchored primarily to the radiation therapy economic analysis by Natesan et al [30]. Workforce optimization used TN(0.30, 0.10; lower=0.01, upper=0.6), informed by scheduling, overtime, and workforce-cost studies [31,32]. For Australia, we used TN(0.38, 0.06) for radiology, TN(0.50, 0.15) for workflow optimization, and TN(0.30, 0.12) for workforce optimization, with the same lower and upper bounds.

We modeled adoption rates with TN priors bounded between 0.01 and 0.35. UK adoption followed TN(0.12, 0.09), and Australian adoption followed TN(0.15, 0.10), informed by national digital health strategy reports and implementation statistics [33-35]. The upper bound of 35% reflects the maximum penetration we considered plausible under existing national AI strategies for 2024; exceeding that level would likely require infrastructure and workforce investments beyond current plans. We also capped effectiveness at 0.6 in radiology and workforce optimization, where human oversight remains substantial, and at 0.8 in workflow optimization, where the automation potential is greater and clinical risk thresholds are lower [33-35].

We specified implementation risk using sector-specific beta distributions. Radiology risks followed Beta(2.3, 1.7), representing a 57% mean risk; workflow optimization used Beta(2.8, 2.2), representing a 56% mean risk; and workforce optimization used Beta(3.5, 3.5), representing a 50% mean risk. These parameters were derived from a systematic review of AI implementation studies, particularly the analysis of deployment failures by Beede et al [17] and an evaluation of clinical integration challenges by Sendak et al [36]. We then further constrained these priors using direct implementation-failure observations in the augmented likelihood. For UK radiology, a study of the NHS AI chest diagnostics program by Ramsay et al [37] reported that 23 of 66 funded NHS trusts had not achieved clinical use at the time of reporting, yielding a failure-rate estimate of 0.348 with measurement uncertainty of 0.059 [37]. Because all 66 trusts had already been funded and committed to deployment, we interpret this as a conditional implementation-failure rate rather than a general market-adoption rate. The Royal College of Radiologists’ 2023 Clinical Radiology Workforce Census reported that 46% of NHS trusts were not using AI tools in radiology practice, which is broadly consistent with that estimate [38]. For workflow optimization and workforce optimization in both countries, and for Australian radiology, where equivalent national deployment surveys were unavailable, we used a pooled estimate. A systematic review of 39 AI randomized controlled trials by Lam et al [39] found that 18 studies, or 46.2%, failed to demonstrate clinically meaningful outcomes. Further, a systematic review of 14 real-world AI implementations by Yin et al [40] found that 6, or 42.9%, did not improve patient outcomes. Inverse-variance pooling of those estimates yielded an implementation-failure anchor (*Y_R_*) of 0.45 with SE (*σ_R_*) of 0.07.

### Sectors Analyzed

We analyzed 3 health care sectors chosen for their differing levels of AI maturity and potential cost impact. Radiology included diagnostic imaging services, with savings anchors derived from breast-screening studies [27-29]. Workflow optimization covered administrative and clinical workflow processes, but in the present implementation, its quantitative savings anchor is the radiation therapy course-level cost reduction reported by Natesan et al [30]. Workforce optimization covered staff scheduling, workload distribution, and broader human resource management, with prior evidence drawn from scheduling, overtime, and workforce-cost studies [31,32].

In each sector, we used country-specific parameters to reflect institutional differences between the United Kingdom’s centralized NHS structure and Australia’s more federated health care system.

### Scenario Analysis

We examined 3 main scenarios to test the sensitivity of our results to implementation conditions, with full results reported in Table S1 in Multimedia Appendix 3. We applied scenario multipliers to 1000 bootstrap samples drawn from approximate posterior distributions, using normal approximations parameterized by posterior means and SDs. The resulting credible intervals (CrIs) therefore represent the 2.5th to 97.5th percentiles of the bootstrap distributions rather than simple transformations of posterior means. Multimedia Appendix 3 also reports a separate 6-scenario temporal analysis—baseline, high inflation, low inflation, currency volatility, technology acceleration, and regulatory constraint—applied to the multiyear projection model. Note that because the interval estimates rely on a normal approximation to the marginal posteriors rather than the raw particle set, this approximation can impose symmetry on skewed distributions and produce boundary effects at the parameter caps.

The baseline scenario used the core posterior distributions. The optimistic scenario assumed enhanced implementation success with reduced risk parameters, for example, Beta(3.3, 2.7) for radiology, representing a 55% mean risk; increased adoption rates at the 75th percentile of the baseline distributions; and effectiveness parameters at the upper quartile. The pessimistic scenario incorporated higher implementation risks, for example, Beta(1.3, 0.7) for radiology, representing a 65% mean risk; reduced adoption rates at the 25th percentile; and lower effectiveness parameters. The conservative scenario increased implementation risk by 10%, reduced adoption by 10%, and reduced effectiveness by 5% relative to baseline. Together, these scenarios span plausible implementation environments while maintaining internal consistency.

### Temporal Projection Parameters and Learning Dynamics

For projections from 2024 to 2030, we defined 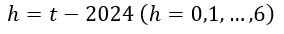 and extended equation 1 as:



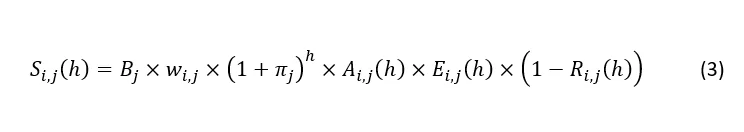



Adoption followed a baseline-preserving logistic learning curve, capped at 35%, with faster uptake assumed in the United Kingdom than in Australia (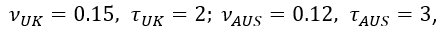, where ν is the maximum proportional gain in adoption above the 2024 baseline and *τ* denotes years since 2024 at the inflection point). Implementation risk decayed geometrically as 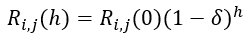 with *δ*=0.05, reflecting organizational learning. Effectiveness increased geometrically as 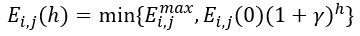 with *γ*=0.03, using caps of 0.60 for radiology and workforce optimization and 0.80 for workflow optimization. Country-specific inflation rates were set at 4% for the United Kingdom and 5% for Australia. Uncertainty was propagated using 1000 Monte Carlo/bootstrap draws from normal approximations to the SMC-derived posterior distributions, parameterized by posterior means and SDs for adoption, implementation risk, effectiveness, and baseline savings. Multimedia Appendix 3 reports the resulting annual and cumulative scenario projections.

### Model Validation

External validation against independent national AI cost-savings benchmarks was not feasible because no published assessment matched the sector-level granularity of our model. In place of formal validation, we conducted face-validity checks by comparing posterior sector savings with analogous published estimates. UK workforce optimization savings of US $591.9 million represent roughly 0.53% of the UK workforce compensation base of about US $111 billion, a magnitude broadly consistent with overtime-reduction studies reporting efficiency gains of 2% to 8% in scheduling-optimization settings [31]. UK radiology savings of US $143.9 million against a radiology base of roughly £2.4 billion (about $3.1 billion) represent about 4.6% of sector spending, which is broadly consistent with the 4% to 7% cost-per-study reductions reported in AI-assisted reading studies [11,27]. These checks support the order-of-magnitude plausibility of our projections, although they do not substitute for external validation against detailed cost-accounting data. Expanding the framework to settings with administrative cost-accounting data would enable formal validation against observed cost trajectories; this is the single most important priority for strengthening these estimates.

All analyses were conducted in MATLAB R2023a using custom SMC functions. A complete summary of Bayesian parameter estimates comparing prior and posterior distributions is provided in Table S2 in Multimedia Appendix 2. The complete MATLAB scripts and SMC implementation code are publicly available (see Data Availability section). All data sources are fully cited and traceable to public repositories (ONS, AIHW, and OECD).

### Ethical Considerations

This study used only publicly available, aggregated health care expenditure data, published peer-reviewed literature, and national health statistics. We did not use patient-level data, personally identifiable information, or clinical records at any stage. Accordingly, no institutional ethics board or Human Research Ethics Committee review was needed. In the United Kingdom, this study falls outside the scope of the UK Policy Framework for Health and Social Care Research [41], which restricts oversight to research involving NHS patients, their tissue, or their identifiable data; computational modeling based on publicly available national statistics does not constitute such research. In Australia, the study was conducted in accordance with the *National Statement on Ethical Conduct in Human Research* [42], which exempts from Human Research Ethics Committee review research that relies solely on publicly available information with no potential for harm to individuals. No human participants were recruited, no clinical interventions were administered, and no informed consent was required.

## Results

### Primary Cost Savings Projections

Our Bayesian analysis revealed substantial potential for AI-driven gross cost savings across both health care systems, with total projected annual gross cost savings of US $948.8 million for the United Kingdom and US $737.1 million for Australia. Table 1 presents the comprehensive posterior estimates for all sectors and countries. The prior-to-posterior evolution of each component for each sector and country is shown in Multimedia Appendix 4.

In the United Kingdom, workforce optimization generated the largest projected savings at US $591.9 million annually (95% CrI US $398.8-US $854.8 million), accounting for 62.4% of total projected UK savings. This result reflects the considerable potential for AI-driven efficiency gains in staff scheduling, workload distribution, and human resource management within the NHS. Workflow optimization contributed US $213.1 million (95% CrI US $133.1-US $300.6 million), representing savings from administrative and clinical process improvement. Radiology contributed US $143.9 million (95% CrI US $111.4-US $169.3 million), despite accounting for a smaller share of total health care expenditure.

Australia showed the same sector ordering but lower absolute savings overall. Workforce optimization generated US $561.4 million annually (95% CrI US $331.9-US $815.3 million), accounting for 76.2% of total projected Australian savings. Workflow optimization contributed US $95.2 million (95% CrI US $62.4-US $135.8 million), and radiology contributed US $80.4 million (95% CrI US $65.3-US $94.9 million). The shared ranking—workforce, workflow, then radiology—suggests that the main cross-country difference lies in scale rather than in the location of savings. At the same time, the CrIs overlap across several sector estimates, so those differences should still be interpreted probabilistically rather than definitively.

**Table 1:** Annual AI cost savings projections by sector and country (2023 US $, millions).

Country	Sector	Mean savings^a^ (95% CrI)^b^	CV^c^ (%)
United Kingdom	Radiology^d^	143.9 (111.4-169.3)	9.4
United Kingdom	Workflow optimization^e^	213.1 (133.1-300.6)	18.2
United Kingdom	Workforce optimization^f^	591.9 (398.8-854.8)	18.2
Australia	Radiology	80.4 (65.3-94.9)	8.8
Australia	Workflow optimization	95.2 (62.4-135.8)	17.6
Australia	Workforce optimization	561.4 (331.9-815.3)	18.6
Total: United Kingdom	All sectors	948.8 (720.6-1173.5)	—^g^
Total: Australia	All sectors	737.1 (526.1-953.6)	—

^a^Posterior estimates of annual AI-driven gross cost savings for the United Kingdom and Australia across 3 health care sectors. Values are reported in 2023 US $, using the International Monetary Fund December 2023 exchange rate for the United Kingdom (£1=US $1.27) and the Reserve Bank of Australia December 2023 exchange rate for Australia (Aus $1=US $0.68).

^b^The 95% credible intervals (CrIs) are the 2.5th to 97.5th percentiles of the raw sequential Monte Carlo (SMC) posterior. The corresponding baseline 95% CrIs in the next table were derived from 1000 bootstrap samples drawn from normal approximations to the SMC-derived posterior and therefore differ slightly (United Kingdom: 718.5-1177.7; Australia: 525.3-950.2). Both representations are internally consistent with their respective sampling procedures.

^c^CV: coefficient of variation of the posterior distribution.

^d^Radiology: diagnostic imaging reading and triage enhancements.

^e^Workflow optimization: administrative and clinical workflow process improvements (evidence base: radiation therapy treatment planning).

^f^Workforce optimization: staff scheduling and human resource management efficiencies.

^g^Not applicable.

### Prior vs Posterior Distribution Evolution

**Figure 1:**
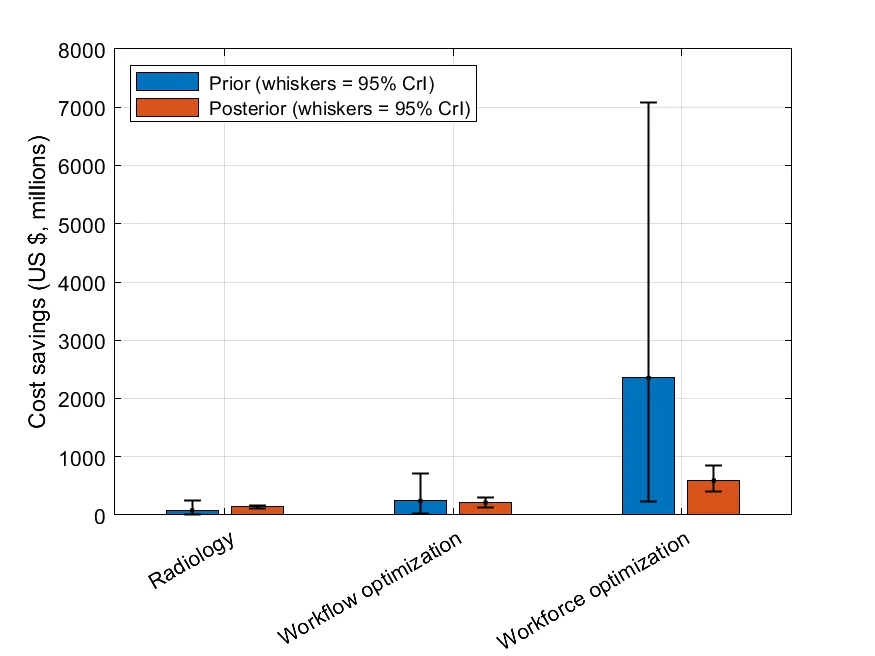
Prior to posterior parameter cost savings by sector in the United Kingdom. CrI: credible interval.

**Figure 2:**
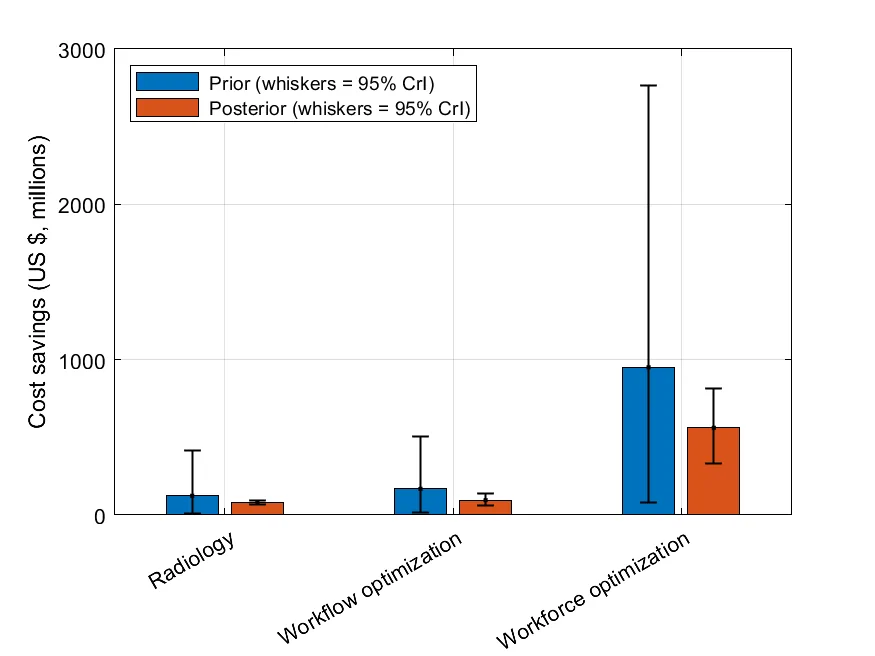
Prior to posterior parameter cost savings by sector in Australia. CrI: credible interval.

Bayesian updating shifted several parameters in meaningful ways relative to their literature-based priors. Figures 1 and 2 illustrate the movement from prior to posterior distributions for the United Kingdom and Australia, respectively, and Table S2 in Multimedia Appendix 2 reports the corresponding numerical estimates.

The clearest change appears in implementation risk. Posterior implementation risk fell below the prior mean in every sector-country pair, indicating that the external failure-rate anchors were more favorable than the literature-derived beta priors. In UK radiology, implementation risk dropped from a prior mean of 57.1% to a posterior mean of 35.5% (95% CrI 24.5%-46.5%), pulled strongly toward the NHS deployment-failure anchor of 0.348 [37]. The remaining 5 sector-country pairs also moved downward, though more modestly: UK workflow fell from 54.5% to 46.8%, UK workforce from 49.8% to 46.4%, Australian radiology from 57.2% to 47.3%, Australian workflow from 55.8% to 47.4%, and Australian workforce from 49.1% to 46.7%. In those cases, the posterior was pulled toward the pooled failure-rate estimate of 0.45 [39,40]. Overall, posterior implementation-risk estimates ranged from 35.5% to 47.4%, uniformly below the prior range of 49.1% to 57.2%. This pattern suggests that the empirical deployment evidence was somewhat more favorable than our conservative prior assumptions, even though implementation risk remained substantial in all sectors and continued to constrain realized savings.

Adoption followed a different pattern. UK radiology adoption rose modestly from 13.6% to 17.0%, consistent with growing uptake of AI tools in chest imaging and mammography. In every other sector-country pair, however, posterior adoption fell below its prior mean. The declines were especially pronounced in workforce optimization, where posterior adoption fell from 13.8% to 3.9% in the United Kingdom and from 16.1% to 10.7% in Australia. These low estimates suggest that organizational barriers to implementation in human resource management are much stronger than national strategy documents alone would imply.

Effectiveness estimates also moved, mainly through shrinkage toward the auxiliary observation values. UK radiology effectiveness was anchored at 43% relative to a prior mean of 43.1%, workflow optimization at 52% relative to 55%, and workforce optimization at 26.8% relative to 30.1%. Australian radiology shifted upward from 36.8% to 42.6%, reflecting the influence of a randomized controlled trial-derived effectiveness anchor. Posterior SDs for effectiveness were also much narrower than prior SDs—for example, in radiology, the SD fell from 10.3% to 2%—showing that the augmented likelihood sharpened those parameter estimates as intended.

### Scenario Analysis Results

Annual scenario analysis showed that projected savings are highly sensitive to implementation conditions. Across scenarios, total projected savings ranged from $356.6 million to $1.845 billion in the UK and from $267.2 million to $1.454 billion in Australia. Table 2 summarizes the sector-by-sector results. Under the optimistic scenario, projected annual savings rose to $1.845 billion in the UK, a 95% increase over the baseline estimate of about $0.949 billion. Australia reached $1.454 billion, a 97% increase over its baseline estimate of about $0.737 billion. These outcomes assumed lower implementation risk, stronger adoption, and effectiveness at the upper quartile of the posterior distributions. In both countries, workforce optimization remained the dominant contributor under these favorable conditions. The optimistic case also makes clear that gains are driven less by any single technology than by system-level conditions—especially adoption and implementation success—that lift all sectors simultaneously.

Under the pessimistic scenario, projected savings fell sharply to US $356.6 million in the United Kingdom and US $267.2 million in Australia. This case assumed implementation risk above 65% across sectors, adoption below 8%, and effectiveness at the lower quartile. The contrast between optimistic and pessimistic scenarios highlights the importance of organizational readiness and implementation success. In both the centralized UK system and the federated Australian system, higher risk and weaker adoption compress projected savings dramatically.

**Figure 3:**
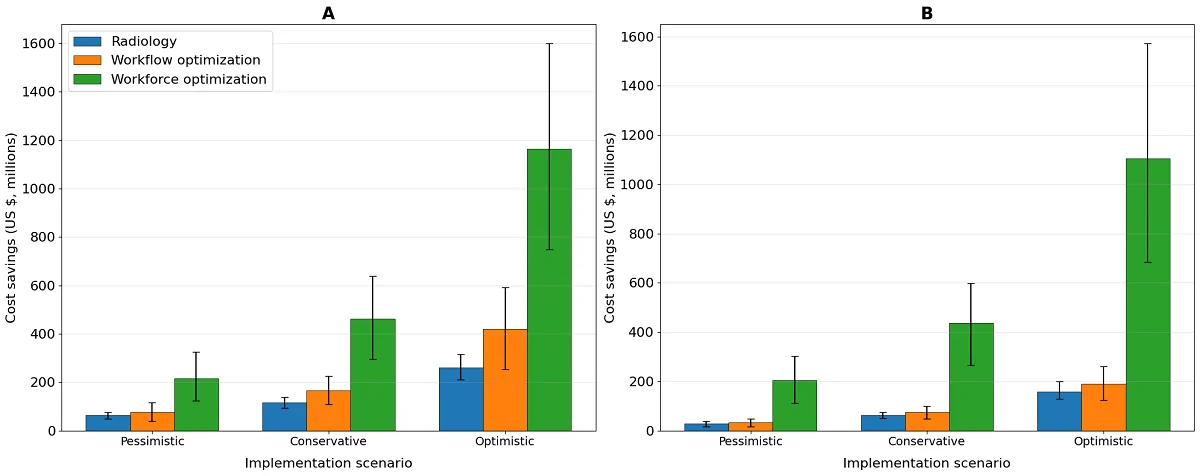
Gross cost saving distribution by sector and implementation scenarios in the United Kingdom and Australia. CrI: credible interval. A: Gross cost savings (United Kingdom): scenario analysis (95% Cris); B: Gross cost savings (Australia): scenario analysis (95% Cris).

The conservative scenario yielded US $744.5 million for the United Kingdom and US $574.6 million for Australia. These estimates may offer the most plausible short-run planning values because they reflect moderate implementation friction rather than best-case or worst-case assumptions. Even so, the corresponding 95% CrIs remained wide, so they are better treated as planning ranges than as precise forecasts—particularly when comparing United Kingdom and Australian totals. Figure 3 illustrates the resulting sectoral distributions, and Tables S1 and S2 in Multimedia Appendix 3 report the annual scenario results together with the separate 2024-2030 temporal scenario analysis.

**Table 2:** Scenario analysis of cost savings by implementation context (US $, millions). Values are posterior means with 95% Bayesian credible intervals (CrIs; 2.5th to 97.5th percentiles of bootstrap distributions drawn from the approximate posterior, including the baseline scenario). Values are expressed in 2023 US $, using the International Monetary Fund December 2023 exchange rate for the United Kingdom (£1=US $1.27) and the Reserve Bank of Australia December 2023 exchange rate for Australia (Aus $1=US $0.68).

Scenario	Country	Radiology^a^ (95% CrI)	Workflow^b^ (95% CrI)	Workforce^c^ (95% CrI)	Total^d^ (95% CrI)
Optimistic^e^	United Kingdom	261.5 (211.7-315.2)	420.2 (253.6-592.8)	1163.6 (748.0-1598.8)	1845.4 (1390.3-2299.0)
Optimistic	Australia	159.2 (128.6-199.5)	188.8 (124.5-259.9)	1105.8 (684.2-1571.3)	1453.8 (1044.7-1935.5)
Pessimistic^f^	United Kingdom	62.8 (47.8-77.0)	77.3 (38.8-115.1)	216.5 (123.2-325.7)	356.6 (252.0-466.9)
Pessimistic	Australia	28.9 (17.2-37.6)	34.1 (17.3-48.2)	204.3 (111.9-302.1)	267.2 (171.1-367.4)
Conservative^g^	United Kingdom	116.3 (93.8-138.1)	166.1 (108.0-226.8)	462.2 (295.6-638.4)	744.5 (570.9-924.7)
Conservative	Australia	62.6 (50.3-74.0)	74.1 (48.7-99.9)	438.0 (266.3-597.7)	574.6 (402.1-739.9)
Baseline^h^	United Kingdom	143.9 (117.1-171.1)	213.1 (135.2-289.3)	591.9 (378.3-799.5)	948.8 (718.5-1177.7)
Baseline	Australia	80.4 (66.4-95.0)	95.2 (61.3-128.4)	561.4 (355.0-766.5)	737.1 (525.3-950.2)

^a^Radiology: diagnostic imaging reading and triage enhancements.

^b^Workflow: administrative and clinical workflow process improvements (evidence base: radiation therapy treatment planning).

^c^Workforce: staff scheduling and human resource management efficiencies.

^d^Totals may not sum exactly because of rounding.

^e^Optimistic scenario: implementation risk reduced to approximately 45%, adoption increased to approximately 17%, and effectiveness at the upper quartile.

^f^Pessimistic scenario: implementation risk >65%, adoption <8%, and effectiveness at the lower quartile.

^g^Conservative scenario: moderate implementation challenges (implementation risk 55% to 60%; adoption approximately 12%).

^h^Baseline scenario: posterior mean estimates from the Bayesian sequential Monte Carlo analysis.

### Cross-Sector Analysis and Total System Impact

Emerging studies suggest that integrating AI across multiple domains—such as radiology, administrative workflows, and workforce planning—may generate meaningful synergies [18,19]. Although our savings function could accommodate such cross-sector effects, we were unable to quantify them because the empirical evidence remains too limited. Even so, the sectoral pattern offers a clear strategic signal. Workforce optimization dominates projected cost savings across scenarios in both countries, contributing 62.4% of UK total cost savings and 76.2% of Australian total cost savings in the baseline case. That pattern suggests that human resource management may be the highest-value target for early AI investment, even though current policy and research attention remain more heavily concentrated on clinical AI applications.

Radiology remains strategically important despite its smaller aggregate savings. In the United Kingdom, it showed the largest prior-to-posterior decline in implementation risk because we were able to anchor the model to direct NHS deployment evidence [37,38]. It may therefore serve as a practical entry point for implementation learning and provide institutional experience that can support later expansion. Workflow optimization also remained economically important in both countries, ranking second in projected savings despite the organizational complexity of administrative and clinical process redesign.

## Discussion

### Principal Findings

Our analysis reveals that AI implementation in the United Kingdom and Australian health care systems has substantial economic potential, with projected annual gross cost savings of US $949 million and US $737 million, respectively. However, 3 critical findings challenge conventional wisdom about health care AI economics. First, workforce optimization dominates potential cost savings across both countries, contributing 62.4% (United Kingdom) and 76.2% (Australia) of total projected benefits despite receiving less attention in AI implementation literature compared to clinical applications. Second, posterior implementation risk falls below prior expectations in every sector (posterior range 35.5%-47.4% vs prior means 49.1%-57.2%) because empirical deployment failure rates are more favorable than the conservative literature-derived beta priors; nevertheless, implementation risk remains the binding constraint on realizing these savings. Third, the substantial variation between pessimistic (US $0.357 and US $0.267 billion for the United Kingdom and Australia, respectively) and optimistic (US $1.845 and US $1.454 billion for the United Kingdom and Australia, respectively) scenarios demonstrates that organizational and implementation factors, rather than technological capabilities, primarily determine AI’s economic impact.

### Comparison With Prior Work

The dominance of workforce optimization cost savings challenges the prevailing focus on clinical AI applications in health care technology assessment. While diagnostic AI in radiology has received extensive research attention and regulatory development, our findings suggest that administrative and human resource applications may offer superior return on investment. This pattern likely reflects substantial inefficiencies in current health care workforce management, where manual scheduling, suboptimal workload distribution, and reactive staffing decisions create opportunities for AI-driven optimization that exceed the efficiency gains available in already streamlined clinical processes.

### Limitations

Several limitations shape how these findings should be interpreted. First, although we defined sector cost categories to be mutually exclusive in order to minimize double counting, some residual overlap cannot be ruled out without more detailed cost-accounting data from individual health systems. Savings estimates should therefore be interpreted as upper bounds on nonoverlapping achievable gains. Second, the sector expenditure weights and observed savings values are constructed from heterogeneous published studies rather than direct measurements of national AI-attributable cost reductions. They represent informed approximations and should not be treated as empirical observations in the classical sense. A deeper consequence of this construction is that 3 latent parameters (*A*, *E*, *R*) are updated from a limited number of constructed savings anchors per sector-country pair—2 in radiology and 1 in workflow and workforce—so the model remains only partially identified and posterior distributions retain sensitivity to prior specifications. A prior sensitivity analysis (Tables S3 and S4 in Multimedia Appendix 3) demonstrates that sector rank orderings and order-of-magnitude estimates are robust to ±20% perturbations of individual prior means and to alternative *σ* scaling factors, but the fundamental identifiability constraint cannot be resolved without larger, sector-specific implementation datasets.

Third, we excluded implementation costs such as software licensing, integration, training, governance, change management, and cybersecurity. Health systems should deduct these costs when translating our projections into business cases. Fourth, most model parameters were derived from published studies conducted in academic medical centers and large hospital networks, which may not represent community hospitals, rural facilities, or lower-resourced settings; transferability across institutions and geographies therefore remains uncertain. Fifth, the model treats adoption rate and implementation risk as statistically independent multiplicative factors. In practice, higher-risk organizations may also adopt AI more slowly, creating a negative correlation that would further reduce savings in high-risk settings. This independence assumption likely introduces a modest upward bias into projected savings. Sixth, several data quality caveats apply collectively: sector expenditure shares are derived from aggregate national health accounts and remain subject to classification uncertainty; cross-country US $ comparisons depend on spot exchange rates; and estimation noise is modeled via sector-specific heteroscedastic priors. Table S3 in Multimedia Appendix 3 reports the sensitivity of posterior savings to the *σ* scaling factor. Seventh, the model does not capture downstream clinical costs that AI deployment may trigger. For example, AI-assisted radiology screening may increase recall rates or biopsy referrals before sensitivity and specificity stabilize in operational settings, generating downstream costs not reflected in our savings estimates. Likewise, workflow AI that accelerates throughput may shift costs to downstream services. Quantifying such second-order effects will require health system-level cost-accounting data linked to AI deployment records.

### Future Directions

Future work could strengthen the framework in several ways. Longitudinal implementation datasets would allow better calibration of adoption curves and risk decay. Natural experiments and richer cost-accounting data could help quantify cross-sector synergies and validate realized savings. The Bayesian framework can be adapted as a decision-support tool for health system planners by parameterizing priors from organization-specific readiness assessments (eg, AI maturity scores, existing digital infrastructure, and workforce capacity) and updating with local deployment data as they accumulate. This would allow progressive narrowing of CrIs over time, transforming the current national-level projections into institution-specific investment guidance. Finally, linking economic outcomes to patient outcomes would provide a fuller assessment of value.

### Conclusion

AI could generate substantial expenditure reductions in both the United Kingdom and Australian health care systems, with projected annual gross cost savings of US $949 million and US $737 million, respectively. In both countries, workforce optimization offers the largest potential gains, while implementation risk remains the main obstacle to realizing them. These findings suggest that policymakers should place as much weight on implementation science, governance, and organizational change as on technology acquisition itself. Health systems may also need to rebalance attention toward workforce optimization, develop stronger implementation-risk assessment frameworks, and create mechanisms for shared learning across centralized and federated systems. The Bayesian framework we present is best understood as a decision-support tool that provides probabilistic planning ranges under uncertainty rather than deterministic forecasts.

## Appendix

Multimedia Appendix 1 Technical model specifications.

Multimedia Appendix 2 Literature synthesis and baseline data.

Multimedia Appendix 3 Extended scenarios, sensitivity analyses, and robustness checks.

Multimedia Appendix 4 Individual parameter distributions.

Multimedia Appendix 5 MATLAB simulation code for replication.

## Abbreviations

AIHW: Australian Institute of Health and Welfare
CrI: credible interval
IMF: International Monetary Fund
MCMC: Markov chain Monte Carlo
NHS: National Health Service
OECD: Organization for Economic Co-operation and Development
ONS: Office for National Statistics
RBA: Reserve Bank of Australia
SMC: sequential Monte Carlo
TN: truncated normal

## Data Availability

All data generated or analyzed during this study are included in this published article and its supplementary information files. The complete MATLAB R2023a scripts and sequential Monte Carlo (SMC) implementation code supporting this study are provided in Multimedia Appendix 5. The package includes (1) BayesianAIHealthcare_Analysis.m—the primary analysis script generating Tables 1 and 2 and Tables S1-S4 in Multimedia Appendix 3; (2) BayesianAIHealthcare_SMCSector.m—the sector-level SMC simulation with augmented log-likelihood; (3) 6 helper files for sensitivity analyses; and (4) a ReadMe file containing instructions to run the simulation code. All prior data included in Multimedia Appendices 1 and 3 are hard coded in the MATLAB scripts. No individual patient data were used or generated.
