# Supplementary material for: Bayesian Analysis of AI-Driven Cost Savings in UK and Australian Health Care Systems: Cross-Sector Implementation Study
Source: JMIR Med Inform. 2026 Aug 26;14:e89113. doi: 10.2196/89113 (PMC13559155; doi:10.2196/89113)
Supplement: Supplementary file 1 [file medinform_v14i1e89113_app1.docx]

**Multimedia Appendix 1: Technical Model Specifications**

This appendix provides detailed technical specifications for the Bayesian simulation model, including complete parameter distributions, healthcare expenditure calculations, and methodological assumptions.

**Table S1: Prior distribution specifications for all model parameters**

| Country | Sector | Parameter | Distribution type | Shape param 1 | Shape param 2 | Lower bound | Upper bound |
| --- | --- | --- | --- | --- | --- | --- | --- |
| UK | Radiology | Adoption Rate | Truncated Normal | 0.12 | 0.09 | 0.01 | 0.35 |
| UK | Radiology | Implementation Risk | Beta | 2.3 | 1.7 | - | - |
| UK | Radiology | Effectiveness | Truncated Normal | 0.46 | 0.13 | 0.01 | 0.60 |
| UK | Workflow Optimization | Adoption Rate | Truncated Normal | 0.12 | 0.09 | 0.01 | 0.35 |
| UK | Workflow Optimization | Implementation Risk | Beta | 2.8 | 2.2 | - | - |
| UK | Workflow Optimization | Effectiveness | Truncated Normal | 0.55 | 0.12 | 0.01 | 0.80 |
| UK | Workforce Optimization | Adoption Rate | Truncated Normal | 0.12 | 0.09 | 0.01 | 0.35 |
| UK | Workforce Optimization | Implementation Risk | Beta | 3.5 | 3.5 | - | - |
| UK | Workforce Optimization | Effectiveness | Truncated Normal | 0.30 | 0.10 | 0.01 | 0.60 |
| AUS | Radiology | Adoption Rate | Truncated Normal | 0.15 | 0.10 | 0.01 | 0.35 |
| AUS | Radiology | Implementation Risk | Beta | 2.3 | 1.7 | - | - |
| AUS | Radiology | Effectiveness | Truncated Normal | 0.38 | 0.128 | 0.01 | 0.60 |
| AUS | Workflow Optimization | Adoption Rate | Truncated Normal | 0.15 | 0.10 | 0.01 | 0.35 |
| AUS | Workflow Optimization | Implementation Risk | Beta | 2.8 | 2.2 | - | - |
| AUS | Workflow Optimization | Effectiveness | Truncated Normal | 0.50 | 0.15 | 0.01 | 0.80 |
| AUS | Workforce Optimization | Adoption Rate | Truncated Normal | 0.15 | 0.10 | 0.01 | 0.35 |
| AUS | Workforce Optimization | Implementation Risk | Beta | 3.5 | 3.5 | - | - |
| AUS | Workforce Optimization | Effectiveness | Truncated Normal | 0.30 | 0.12 | 0.01 | 0.60 |

**Table S2: Literature source mapping and rationale**

| Country-sector-parameter | Mathematical specification | Primary literature source | Distribution bounds rationale |
| --- | --- | --- | --- |
| UK-Radiology-Adoption Rate | Normal (μ=0.12, σ=0.09) | **Reference:** Department for Digital, Culture, Media & Sport. National AI Strategy: AI Action Plan. UK Government; July 2022. **URL:** <https://www.gov.uk/government/publications/national-ai-strategy-ai-action-plan/national-ai-strategy-ai-action-plan> | UK AI adoption rate in healthcare; bounds ensure realistic adoption range |
| UK-Radiology-Implementation Risk | Beta (α=2.3, β=1.7) | **1.** NHS England. Artificial Intelligence Deployment Platform Pilot - Privacy Notice. NHS England; 2024. **URL:** <https://www.england.nhs.uk/contact-us/privacy-notice/artificial-intelligence-deployment-platform-pilot/>  **2.** Wolff J, Pauling J, Keck A, Baumbach J. The economic impact of artificial intelligence in health care: systematic review. *J Med Internet Res*. 2020;22(2):e16866. **DOI:** 10.2196/16866 **URL:** <https://www.jmir.org/2020/2/e16866/> | NHS radiology AI pilot failure rate ~58%; bounds [0,1] for probability |
| UK-Radiology-Effectiveness | Normal (μ=0.46, σ=0.13) | **1.** Lång K, Josefsson V, Larsson AM, et al. Artificial intelligence-supported screen reading versus standard double reading in the Mammography Screening with Artificial Intelligence trial (MASAI): a clinical safety analysis of a randomised, controlled, non-inferiority, single-blinded, screening accuracy study. Lancet Oncol. 2023;24(8):936-944.  **DOI:** [10.1016/S1470-2045(23)00298-X](https://doi.org/10.1016/S1470-2045(23)00298-X) **2.** Marinovich ML, Houssami N, Macaskill P, et al. Breast cancer screening using tomosynthesis or mammography: a meta-analysis of cancer detection and recall. *J Natl Cancer Inst*. 2018;110(9):942-949. **DOI:** [10.1093/jnci/djy121](https://doi.org/10.1093/jnci/djy121)  **URL:** <https://academic.oup.com/jnci/article/110/9/942/5068658> | Weighted mean from breast cancer screening studies; 60% upper bound for clinical feasibility |
| UK-Workflow Optimization-Adoption Rate | Normal (μ=0.12, σ=0.09) | **1.** Chen JH, Asch SM. Machine learning and prediction in medicine - beyond the peak of inflated expectations. *N Engl J Med*. 2017;376(26):2507-2509. **DOI:** 10.1056/NEJMp1702071 **URL:** <https://www.nejm.org/doi/10.1056/NEJMp1702071>  **2.** Devaraju S, Boyd T. AI-augmented workforce scheduling in cloud-enabled environments. *World Journal of Advanced Research and Reviews*. 2021;12(3):674-680. **DOI:** [10.30574/wjarr.2021.12.3.0691](https://doi.org/10.30574/wjarr.2021.12.3.0691)  **URL:** <https://wjarr.com/sites/default/files/WJARR-2021-0691.pdf> | Consistent with radiology adoption; workflow complexity may limit uptake |
| UK-Workflow Optimization-Implementation Risk | Beta (α=2.8, β=2.2) | 1. NHS England. Artificial Intelligence Deployment Platform Pilot - Privacy Notice. NHS England; 2024.  URL: https://www.england.nhs.uk/contact-us/privacy-notice/artificial-intelligence-deployment-platform-pilot/.  2. Natesan D, Eisenstein EL, Thomas SM, Eclov NCW, Dalal NH, Stephens SJ, Malicki M, Shields S, Cobb A, Mowery YM, Niedzwiecki D, Tenenbaum JD, Palta M, Hong JC. Health Care Cost Reductions with Machine Learning-Directed Evaluations during Radiation Therapy - An Economic Analysis of a Randomized Controlled Study. NEJM AI. 2024;1(4). doi:10.1056/aioa2300118 | Workflow optimization has higher success rate ~56%; more mature technology |
| UK-Workflow Optimization-Effectiveness | Normal (μ=0.55, σ=0.12) | Natesan D, Eisenstein EL, Thomas SM, Eclov NCW, Dalal NH, Stephens SJ, Malicki M, Shields S, Cobb A, Mowery YM, Niedzwiecki D, Tenenbaum JD, Palta M, Hong JC. Health Care Cost Reductions with Machine Learning-Directed Evaluations during Radiation Therapy - An Economic Analysis of a Randomized Controlled Study. NEJM AI. 2024;1(4). doi:10.1056/aioa2300118 | Higher effectiveness for workflow tasks; 80% upper bound per workflow effectiveness cap (Equation 3) |
| UK-Workforce Optimization-Adoption Rate | Normal (μ=0.12, σ=0.09) | Department for Digital, Culture, Media & Sport. National AI Strategy: AI Action Plan. UK Government; July 2022. **URL:** <https://www.gov.uk/government/publications/national-ai-strategy-ai-action-plan/national-ai-strategy-ai-action-plan> | Conservative adoption due to workforce resistance and training requirements |
| UK-Workforce Optimization-Implementation Risk | Beta (α=3.5, β=3.5) | **1.** Chen JH, Asch SM. Machine learning and prediction in medicine - beyond the peak of inflated expectations. *N Engl J Med*. 2017;376(26):2507-2509. **DOI:** 10.1056/NEJMp1702071 **URL:** <https://www.nejm.org/doi/10.1056/NEJMp1702071>  **2.** Devaraju S, Boyd T. AI-augmented workforce scheduling in cloud-enabled environments. *World Journal of Advanced Research and Reviews*. 2021;12(3):674-680. **DOI:** [10.30574/wjarr.2021.12.3.0691](https://doi.org/10.30574/wjarr.2021.12.3.0691)  **URL:** <https://wjarr.com/sites/default/files/WJARR-2021-0691.pdf> | Balanced risk profile ~50%; workforce changes require organizational alignment |
| UK-Workforce Optimization-Effectiveness | Normal (μ=0.30, σ=0.10) | **1.** Chen JH, Asch SM. Machine learning and prediction in medicine - beyond the peak of inflated expectations. *N Engl J Med*. 2017;376(26):2507-2509. **DOI:** 10.1056/NEJMp1702071 **URL:** <https://www.nejm.org/doi/10.1056/NEJMp1702071>  **2.** Devaraju S, Boyd T. AI-augmented workforce scheduling in cloud-enabled environments. *World Journal of Advanced Research and Reviews*. 2021;12(3):674-680. **DOI:** [10.30574/wjarr.2021.12.3.0691](https://doi.org/10.30574/wjarr.2021.12.3.0691)  **URL:** <https://wjarr.com/sites/default/files/WJARR-2021-0691.pdf> | Meta-analysis shows 27-32% cost reduction; conservative upper bound for sustainability |
| AUS-Radiology-Adoption Rate | Normal (μ=0.15, σ=0.10) | Australian Government Department of Industry, Science and Resources. National Framework for the Assurance of Artificial Intelligence in Government. June 2024. **URL:** <https://www.finance.gov.au/government/public-data/data-and-digital-ministers-meeting/national-framework-assurance-artificial-intelligence-government> | Slightly higher adoption due to National AI Strategy; similar bounds as UK |
| AUS-Radiology-Implementation Risk | Beta (α=2.3, β=1.7) | **1.** Royal Australian and New Zealand College of Radiologists. RANZCR Submission to the Select Committee on Adopting Artificial Intelligence (AI). May 2024. **URL:** <https://www.aph.gov.au/DocumentStore.ashx?id=656d5dba-052c-49e2-bd98-baa98b7515c1&subId=756126>  2. Beede E, Baylor E, Hersch F, et al. A human-centered evaluation of a deep learning system deployed in clinics for the detection of diabetic retinopathy. *Proc ACM Conf Hum Factor Comput Syst*. 2020;2020:1-12. **DOI:** 10.1145/3313831.3376718 **URL:** <https://dl.acm.org/doi/10.1145/3313831.3376718> | Similar risk profile to UK radiology; fragmented governance increases failure risk |
| AUS-Radiology-Effectiveness | Normal (μ=0.38, σ=0.128) | **1.** Lång K, Hofvind S, Rodríguez-Ruiz A, et al. Artificial intelligence-supported screen reading versus standard double reading in the Mammography Screening with Artificial Intelligence trial (MASAI): a clinical safety analysis of a randomised, controlled, non-inferiority, single-blinded screening accuracy study. Lancet Oncol. 2023;24(8):936-944.  DOI: [10.1016/S1470-2045(23)00298-X](https://doi.org/10.1016/S1470-2045(23)00298-X)  **URL:** <https://www.thelancet.com/journals/lanonc/article/PIIS1470-2045(23)00298-X/abstract>  **2.** Australian Institute of Health and Welfare. BreastScreen Australia monitoring report 2023. AIHW; 2023. **URL:** <https://nla.gov.au/nla.obj-3242546687/view> | Slightly lower effectiveness due to different screening protocols; same upper bound |
| AUS-Workflow Optimization-Adoption Rate | Normal (μ=0.15, σ=0.10) | Australian Government Department of Industry, Science and Resources. National Framework for the Assurance of Artificial Intelligence in Government. June 2024. **URL:** <https://www.finance.gov.au/government/public-data/data-and-digital-ministers-meeting/national-framework-assurance-artificial-intelligence-government> | Consistent with radiology adoption; federal coordination may facilitate uptake |
| AUS-Workflow Optimization-Implementation Risk | Beta (α=2.8, β=2.2) | **1.** Scott IA, van der Vegt A, Canaris S, Nolan P, Pointon K. Preparing healthcare organisations for using artificial intelligence effectively. *Aust Health Rev*. 2025;49:AH25102. **doi:**[**10.1071/AH25102**](https://doi.org/10.1071/AH25102)  **2.** van Dooijeweert, C., Flach, R.N., ter Hoeve, N.D. *et al.* Clinical implementation of artificial-intelligence-assisted detection of breast cancer metastases in sentinel lymph nodes: the CONFIDENT-B single-center, non-randomized clinical trial. *Nat Cancer* **5**, 1195–1205 (2024). <https://doi.org/10.1038/s43018-024-00788-z> | Similar to UK workflow risk, but calibrated using Australian implementation conditions and informed by CONFIDENT-B as a real-world Australian deployment analogue for implementation friction rather than as a workflow-specific effectiveness study. |
| AUS-Workflow Optimization-Effectiveness | Normal (μ=0.50, σ=0.15) | Natesan D, Eisenstein EL, Thomas SM, Eclov NCW, Dalal NH, Stephens SJ, Malicki M, Shields S, Cobb A, Mowery YM, Niedzwiecki D, Tenenbaum JD, Palta M, Hong JC. Health Care Cost Reductions with Machine Learning-Directed Evaluations during Radiation Therapy - An Economic Analysis of a Randomized Controlled Study. NEJM AI. 2024;1(4). doi:10.1056/aioa2300118  Royal Australian and New Zealand College of Radiologists. RANZCR Submission to the Select Committee on Adopting Artificial Intelligence (AI). May 2024.  URL: https://www.aph.gov.au/DocumentStore.ashx?id=656d5dba-052c-49e2-bd98-baa98b7515c1&subId=756126 | Moderate effectiveness; higher uncertainty due to diverse state systems |
| AUS-Workforce Optimization-Adoption Rate | Normal (μ=0.15, σ=0.10) | Australian Government Department of Industry, Science and Resources. National Framework for the Assurance of Artificial Intelligence in Government. June 2024. **URL:** <https://www.finance.gov.au/government/public-data/data-and-digital-ministers-meeting/national-framework-assurance-artificial-intelligence-government> | Federal leadership may accelerate workforce AI adoption compared to UK |
| AUS-Workforce Optimization-Implementation Risk | Beta (α=3.5, β=3.5) | **1.** Scott IA, van der Vegt A, Canaris S, Nolan P, Pointon K. Preparing healthcare organisations for using artificial intelligence effectively. *Aust Health Rev*. 2025;49:AH25102. **doi:**[**10.1071/AH25102**](https://doi.org/10.1071/AH25102)  **2.** Chen JH, Asch SM. Machine learning and prediction in medicine - beyond the peak of inflated expectations. *N Engl J Med*. 2017;376(26):2507-2509. **DOI:** 10.1056/NEJMp1702071 **URL:** <https://www.nejm.org/doi/10.1056/NEJMp1702071> | Balanced risk; federal-state coordination challenges similar to UK organizational issues |
| AUS-Workforce Optimization-Effectiveness | Normal (μ=0.30, σ=0.12) | **1.** Chen JH, Asch SM. Machine learning and prediction in medicine - beyond the peak of inflated expectations. *N Engl J Med*. 2017;376(26):2507-2509. **DOI:** 10.1056/NEJMp1702071 **URL:** <https://www.nejm.org/doi/10.1056/NEJMp1702071>  **2.** Australian Institute of Health and Welfare. BreastScreen Australia monitoring report 2023. AIHW; 2023. **URL:** <https://nla.gov.au/nla.obj-3242546687/view> | Lower baseline effectiveness, with a conservative Australian calibration; the AIHW BreastScreen Australia monitoring report is retained as contextual operational evidence rather than as a direct workforce-effectiveness study. |

*Notes*: Beta distributions used for implementation risk parameters (bounded 0-1). Truncated normal distributions for adoption rates and effectiveness parameters. All parameters updated through Bayesian inference with Sequential Monte Carlo sampling.

**Table S3: Healthcare expenditure base values**

| Country | Sector | Value | Unit | Source |
| --- | --- | --- | --- | --- |
| UK | **Total healthcare** | 292 | GBP billion | ONS 2023 |
| UK | Radiology | 0.83% | of total | NHS/Kalidindi |
| UK | Workflow optimization | 2.0% | of total | DHSC 2020 |
| UK | Workforce optimization | 30% | of total | NHS 2024 |
|  |  |  |  |  |
| AUS | **Total healthcare** | 252.5 | AUD billion | AIHW 2023 |
| AUS | Radiology | 2.8% | of total | AIHW imaging |
| AUS | Workflow optimization | 2.77% | of total | AIHW admin |
| AUS | Workforce optimization | 52.9% | proxy share applied to  total healthcare expenditure | AIHW hospitals |

**Sector Weight Calculations:**

**UK Radiology (0.83%):**

NHS imaging services: £2.0-2.5 billion (Kalidindi & Gandhi, 2023)

Estimated total UK radiology: £2.35-2.5 billion

Weight = £2.4B / £292B = 0.83%

**UK Workflow Optimization (2.0%):**

Workflow expense: £5.84 billion (2% of £292B)

A sector weight of 2.0% of total UK healthcare expenditure is consistent with the Department of Health and Social Care’s (DHSC) ‘Busting Bureaucracy’ report, which indicates that administration represents approximately 2% of healthcare spending in the UK. Using total healthcare expenditure of £292 billion, this yields an estimated workflow-related expenditure base of £5.84 billion for the UK workflow optimization sector. The DHSC document is available at: <https://www.gov.uk/government/calls-for-evidence/reducing-bureaucracy-in-the-health-and-social-care-system-call-for-evidence/outcome/busting-bureaucracy-empowering-frontline-staff-by-reducing-excess-bureaucracy-in-the-health-and-care-system-in-england>.

**UK Workforce Optimization (30%):**

NHS staff costs: £81.7 billion (2023/24)

Represents ~28-32% of total healthcare spending

Includes salaries, pensions, training, locum costs

**Australia Radiology (2.8%):**

Medicare-subsidized imaging: $5.3 billion AUD

Plus out-of-pocket and private: $1.5-2 billion AUD

Total estimate:$7.0 billion AUD

Weight =$7.0B /$252.5B = 2.8%

**Australia Workflow Optimization (2.77%):**

Healthcare administration:

$7.0 billion AUD (AIHW 2023)

Weight =$7.0B /$252.5B = 2.77%

**Australia Workforce Optimization (22.4% of total healthcare expenditure; 52.9% of recurrent public hospital expenditure):**

Public hospital workforce expenditure: AUD 56.62 billion

Represents 52.9% of recurrent public hospital expenditure

Effective model weight = AUD 56.62B / AUD 252.5B (total healthcare expenditure) = 22.4% of total healthcare expenditure

**Exchange Rate Specifications:**

UK: £1 =$1.27 USD (IMF, December 2023)

Australia: A$1 =$0.68 USD (RBA, December 2023)

**Inflation Adjustment:**

All values expressed in 2023 constant prices

No adjustment needed as base year is 2023

Future projections will use country-specific inflation rates
