# Supplementary material for: Bayesian Analysis of AI-Driven Cost Savings in UK and Australian Health Care Systems: Cross-Sector Implementation Study
Source: JMIR Med Inform. 2026 Aug 26;14:e89113. doi: 10.2196/89113 (PMC13559155; doi:10.2196/89113)
Supplement: Supplementary file 2 [file medinform_v14i1e89113_app2.docx]

**Multimedia Appendix 2: Literature Synthesis and Baseline Data**

**Table S1: Prior cost-saving data from Literature**

| **Domain** | **Implementation context** | **Annual savings ($US, millions)** | **Conversion method** |
| --- | --- | --- | --- |
| **Radiology** | Breast cancer screening (AI triaging)  Lång et al. (2023) [27] | **UK: $150.62; AU:** **$82.84**  N = 80,020 | • *UK: 2M screens/year. Savings: 2M × $170/screen × 44.3% = $150.62M.*  • *AU: 1.1M screens/year. Savings: 1.1M screens × $170 × 44.3% = $82.84M.* • *Baseline mammogram costs: $250/scan (CDC 2023)* • *NHS screens cost £62-83/exam (~$79-105).*  • *For comparability across the two radiology anchor studies, the conversion formulas use a standardized unit screening cost of US$170 per screen for both countries.* |
| **Radiology** | Breast cancer screening (AI as independent reader, BreastScreen AU) [29]; Marinovich et al. (2018) [28]. | **UK: $140.8  AU: $77.4**  N**=**108,970 | • UK: 2M screens × $170 × 41.4% = **$140.8 mil**  • AU: 1.1M × $170 × 41.4% = **$77.4 mil** |
| **Workflow** | ML-directed patient evaluations during radiation therapy; 52% cost reduction per course (95% CI: $1,450–$1,783, *p* = 0.03); Natesan et al. (2024) [30] | **UK: $210.1**  **AU: $93.7**  N=311 radiation therapy courses (305 patients) | • UK: 130,000 NHS annual radiotherapy courses × $1,616/course = $210.1 mil  AU: 58,000 AUS annual radiotherapy courses × $1,616/course = $93.7 mil |
| **Workforce** | AI-driven nurse staffing optimization; 27.2% overtime cost reduction (95% CI: 18.22–36.18%), *n* **=** 15,000 shifts  Chen et al. (2024) [31] | **UK: $581.25 AU: $554.54** | • UK: (4,745 clinical consultants × $188,595 + 16,804 Radiographers and Support staff/Nurses × $73,914) x 0.272 = **$581.25 mil**.  • AU: (2,411 Radiologists × $261,800 + 16,693 Radiographers and Support staff/Nurses × $84,320) × 0.272 = **$554.54 mil**.  • UK: Clinical consultant salary (including on-cost of 1.344%): $188,595; Radiographers and Support staff/Nurses salary $73,914 (including on-cost of 1.344%) AU: Radiologist salary (including on-cost of 1.175%): $261,800; Radiographers and Support staff/Nurses salary $84,320 (including on-cost of 1.175%) |

Note: The following exchange rates were applied for converting the British and Australian currencies into US dollars: UK: £1 = $1.27; AU: AU$1 = $0.68 (IMF December 2023 for UK; RBA December 2023 for Australia). The cost-saving figures are arrived at assuming 2 million annual breast screenings in the UK and 1.1 million in Australia at a cost of USD $170/screen. For example, according to Lång et al. (2023), cost saving in the UK Radiology: 2M screens/year × $170/screen × 44.3% cost saving = $150.62 million cost saving/year. The second cost-saving measure in radiology (Marinovich et al., 2018) aligns with NHS and Australian costing.

**Table S2: Bayesian Parameter Estimates**

| **Country** | **Sector** | **Parameter** | **Distribution** | **Mean** | **Median** | **Std Dev** | **Lower 95CI** | **Upper 95CI** |
| --- | --- | --- | --- | --- | --- | --- | --- | --- |
| **UK** | **Radiology** | Adoption Rate | Prior | 0.14 | 0.13 | 0.07 | 0.02 | 0.31 |
| **UK** | **Radiology** | Adoption Rate | Posterior | 0.170 | 0.170 | 0.023 | 0.125 | 0.215 |
| **UK** | **Radiology** | Implementation Risk | Prior | 0.57 | 0.59 | 0.22 | 0.13 | 0.94 |
| **UK** | **Radiology** | Implementation Risk | Posterior | 0.355 | 0.355 | 0.056 | 0.245 | 0.465 |
| **UK** | **Radiology** | Effectiveness | Prior | 0.43 | 0.45 | 0.10 | 0.20 | 0.59 |
| **UK** | **Radiology** | Effectiveness | Posterior | 0.430 | 0.430 | 0.020 | 0.391 | 0.469 |
| **UK** | **Radiology** | Cost Savings (mil.) | Prior | 76.59 | 60.03 | 64.92 | 4.45 | 254.95 |
| **UK** | **Radiology** | **Cost Savings (mil.)** | Posterior | 143.90 | 143.90 | 13.53 | 111.40 | 169.30 |
| **UK** | **Workflow Optimization** | Adoption Rate | Prior | 0.14 | 0.13 | 0.08 | 0.02 | 0.31 |
| **UK** | **Workflow Optimization** | Adoption Rate | Posterior | 0.106 | 0.106 | 0.024 | 0.059 | 0.153 |
| **UK** | **Workflow Optimization** | Implementation Risk | Prior | 0.56 | 0.57 | 0.21 | 0.16 | 0.92 |
| **UK** | **Workflow Optimization** | Implementation Risk | Posterior | 0.468 | 0.468 | 0.069 | 0.333 | 0.603 |
| **UK** | **Workflow Optimization** | Effectiveness | Prior | 0.55 | 0.55 | 0.11 | 0.33 | 0.75 |
| **UK** | **Workflow Optimization** | Effectiveness | Posterior | 0.520 | 0.520 | 0.028 | 0.465 | 0.575 |
| **UK** | **Workflow Optimization** | Cost Savings (mil.) | Prior | 253.87 | 202.12 | 197.68 | 20.55 | 755.54 |
| **UK** | **Workflow Optimization** | **Cost Savings (mil.)** | Posterior | 213.10 | 213.10 | 38.78 | 133.10 | 300.60 |
| **UK** | **Workforce Optimization** | Adoption Rate | Prior | 0.13 | 0.13 | 0.07 | 0.02 | 0.29 |
| **UK** | **Workforce Optimization** | Adoption Rate | Posterior | 0.039 | 0.039 | 0.011 | 0.017 | 0.061 |
| **UK** | **Workforce Optimization** | Implementation Risk | Prior | 0.50 | 0.49 | 0.18 | 0.16 | 0.86 |
| **UK** | **Workforce Optimization** | Implementation Risk | Posterior | 0.464 | 0.464 | 0.064 | 0.339 | 0.589 |
| **UK** | **Workforce Optimization** | Effectiveness | Prior | 0.30 | 0.31 | 0.10 | 0.11 | 0.50 |
| **UK** | **Workforce Optimization** | Effectiveness | Posterior | 0.268 | 0.268 | 0.042 | 0.186 | 0.350 |
| **UK** | **Workforce Optimization** | Cost Savings (mil.) | Prior | 2304.37 | 1901.10 | 1719.20 | 241.67 | 6656.14 |
| **UK** | **Workforce Optimization** | **Cost Savings (mil.)** | Posterior | 591.90 | 591.90 | 107.73 | 398.80 | 854.80 |
| **AUS** | **Radiology** | Adoption Rate | Prior | 0.16 | 0.16 | 0.08 | 0.03 | 0.32 |
| **AUS** | **Radiology** | Adoption Rate | Posterior | 0.076 | 0.076 | 0.013 | 0.052 | 0.102 |
| **AUS** | **Radiology** | Implementation Risk | Prior | 0.57 | 0.58 | 0.22 | 0.13 | 0.92 |
| **AUS** | **Radiology** | Implementation Risk | Posterior | 0.473 | 0.473 | 0.070 | 0.337 | 0.603 |
| **AUS** | **Radiology** | Effectiveness | Prior | 0.37 | 0.37 | 0.12 | 0.13 | 0.57 |
| **AUS** | **Radiology** | Effectiveness | Posterior | 0.426 | 0.426 | 0.020 | 0.384 | 0.458 |
| **AUS** | **Radiology** | Cost Savings (mil.) | Prior | 121.17 | 92.03 | 104.09 | 6.84 | 394.03 |
| **AUS** | **Radiology** | **Cost Savings (mil.)** | Posterior | 80.40 | 80.40 | 7.33 | 65.30 | 94.90 |
| **AUS** | **Workflow Optimization** | Adoption Rate | Prior | 0.16 | 0.16 | 0.08 | 0.03 | 0.32 |
| **AUS** | **Workflow Optimization** | Adoption Rate | Posterior | 0.075 | 0.075 | 0.017 | 0.042 | 0.108 |
| **AUS** | **Workflow Optimization** | Implementation Risk | Prior | 0.56 | 0.57 | 0.20 | 0.19 | 0.92 |
| **AUS** | **Workflow Optimization** | Implementation Risk | Posterior | 0.474 | 0.474 | 0.067 | 0.336 | 0.606 |
| **AUS** | **Workflow Optimization** | Effectiveness | Prior | 0.49 | 0.50 | 0.14 | 0.21 | 0.76 |
| **AUS** | **Workflow Optimization** | Effectiveness | Posterior | 0.518 | 0.518 | 0.027 | 0.467 | 0.569 |
| **AUS** | **Workflow Optimization** | Cost Savings (mil.) | Prior | 236.72 | 184.08 | 185.46 | 22.26 | 722.00 |
| **AUS** | **Workflow Optimization** | **Cost Savings (mil.)** | Posterior | 95.20 | 95.20 | 16.50 | 62.40 | 135.80 |
| **AUS** | **Workforce Optimization** | Adoption Rate | Prior | 0.16 | 0.16 | 0.08 | 0.02 | 0.31 |
| **AUS** | **Workforce Optimization** | Adoption Rate | Posterior | 0.107 | 0.107 | 0.030 | 0.048 | 0.166 |
| **AUS** | **Workforce Optimization** | Implementation Risk | Prior | 0.50 | 0.50 | 0.18 | 0.17 | 0.84 |
| **AUS** | **Workforce Optimization** | Implementation Risk | Posterior | 0.467 | 0.467 | 0.065 | 0.329 | 0.599 |
| **AUS** | **Workforce Optimization** | Effectiveness | Prior | 0.30 | 0.30 | 0.11 | 0.08 | 0.52 |
| **AUS** | **Workforce Optimization** | Effectiveness | Posterior | 0.268 | 0.268 | 0.044 | 0.178 | 0.354 |
| **AUS** | **Workforce Optimization** | Cost Savings (mil.) | Prior | 1396.02 | 1093.43 | 1078.11 | 101.29 | 4114.10 |
| **AUS** | **Workforce Optimization** | **Cost Savings (mil.)** | Posterior | 561.40 | 561.40 | 104.69 | 331.90 | 815.30 |

**Sequential Monte Carlo (SMC) algorithm details**

The Sequential Monte Carlo (SMC) approach was selected over traditional Markov Chain Monte Carlo (MCMC) methods as it requires minimal tuning and is well-suited to analyzing multiple datasets. The algorithm generates posterior distributions by sampling *N* = 1,000 particles from a sequence of distributions that connect the prior and the posterior, accomplished by annealing the likelihood function.

**Table S3: Key algorithmic steps:**

| **Step #** | **Process** | **Description** |
| --- | --- | --- |
| 1. | **Annealing** | At each iteration of the SMC algorithm, the next temperature in the annealing schedule is selected such that the effective sample size (ESS) is approximately 500. |
| 2. | **Resampling** | A resampling step is performed to eliminate particles with low importance weights and duplicate those with high importance weights. |
| 3. | **Perturbation** | A Markov chain Monte Carlo (MCMC) kernel is applied to each resampled particle to perturb it. This step reduces particle duplication and enhances particle diversity. |
| 4. | **Adaptation** | The number of MCMC iterations is determined adaptively based on an estimate of the MCMC acceptance rate. |

This process ensured robust performance, with the number of unique particles consistently remaining above 900 (90%) throughout the analysis.
