# Supplementary material for: Bayesian Analysis of AI-Driven Cost Savings in UK and Australian Health Care Systems: Cross-Sector Implementation Study
Source: JMIR Med Inform. 2026 Aug 26;14:e89113. doi: 10.2196/89113 (PMC13559155; doi:10.2196/89113)
Supplement: Supplementary file 3 [file medinform_v14i1e89113_app3.docx]

**Multimedia Appendix 3: Extended Scenarios, Sensitivity Analyses and Robustness Checks**

**Scenario Factor Specifications:**

Multimedia Appendix 3 reports two distinct scenario sets. Table S1 presents the annual implementation scenarios used in the main text – Baseline, Optimistic, Pessimistic, and Conservative – whereas Table S2 presents the separate 2024–2030 temporal scenarios: Baseline, High inflation, Low inflation, Currency volatility, Tech acceleration, and Regulatory constraint. The adjustment factors listed below apply to the temporal scenario set reported in Table S2. All savings values in Table S2 incorporate country-specific healthcare inflation (UK 4.0%, Australia 5.0% per annum) and are expressed in nominal USD; values in Tables 1, 2, and Table S1 are in constant 2023 USD.

- Baseline: No adjustments (inflation multiplier =1.0, adoption multiplier =1.0, risk multiplier =1.0, effectiveness multiplier =1.0)
- High inflation: 50% higher inflation impact (inflation multiplier =1.5), with reduced adoption (×0.95) and effectiveness (×0.95), increased risk (×1.1)
- Low inflation: 30% lower inflation impact (inflation multiplier =0.7), with enhanced adoption (×1.05) and effectiveness (×1.02), reduced risk (×0.95)
- Currency volatility: Standard inflation, reduced adoption (×0.9) and effectiveness (×0.98), increased risk (×1.15)
- Tech acceleration: Enhanced adoption (×1.3) and effectiveness (×1.25), reduced risk (×0.8)
- Regulatory constraint: Reduced adoption (×0.7) and effectiveness (×0.85), increased risk (×1.4)

**Table S1: Extended scenario analysis**

| Scenario | Country | Total Savings ($US mil.) | Risk level | Implementation probability |
| --- | --- | --- | --- | --- |
| Baseline | **UK** | 948.8 | Medium (50-55%) | Medium (65-75%) |
| Baseline | **AUS** | 737.1 | Medium (50-55%) | Medium (65-75%) |
| Optimistic | **UK** | 1,845.4 | Low (45-50%) | High (75-85%) |
| Optimistic | **AUS** | 1,453.8 | Low (45-50%) | High (75-85%) |
| Pessimistic | **UK** | 356.6 | High (65-70%) | Low (25-35%) |
| Pessimistic | **AUS** | 267.2 | High (65-70%) | Low (25-35%) |
| Conservative | **UK** | 744.5 | Medium (55-60%) | Medium (60-70%) |
| Conservative | **AUS** | 574.6 | Medium (55-60%) | Medium (60-70%) |

**Temporal Projection Methodology (2024-2030)**

**Projection Framework:** The temporal projections from 2024 to 2030 use *h* to index elapsed years since 2024 ($h=0,\ldots,6$) and apply the same time-varying parameterization as Equation (3) in the main text.

1. Learning Curve Effects:

- Adoption rates follow a baseline-preserving logistic learning curve:

$A_{ij}\left( h \right)=A_{ij}(0)\left[ 1+\nu_{j}\left( \frac{1}{1+e^{-\left( h-\tau_{j} \right)}}-\frac{1}{1+e^{\tau_{j}}} \right) \right]$

- UK: $\nu_{UK}=0.15$, $\tau_{UK}=2$ (inflection point);

- Australia: $\nu_{AUS}=0.12$, $\tau_{AUS}=3$ (delayed inflection) where $\sigma₀=\sigma\left( -h_{\beta} \right)=1/\left( 1 + exp\left( h_{\beta} \right) \right)$ is the logistic value at $h = 0$, ensuring $A\left( 0 \right) = A_{base}$ (baseline-preserving). UK: $h_{\beta} = 2,\sigma₀ \approx0.119$; AUS: $h_{\beta} = 3,\sigma₀ \approx0.047$.

2. Implementation Risk Decay:

- Risk parameters decline geometrically as experience accumulates:

$R_{ij}\left( h \right)=R_{ij}{(0)\left( 1-\delta\right)}^{h}$, where $\delta=0.05$ annually

- Reflects organizational learning and technology maturation

3. Effectiveness Improvements:

- AI effectiveness grows geometrically with data accumulation and algorithm refinement:

- $E_{ij}\left( h \right)=\min\{E_{ij}\left( 1+\gamma\right)^{h}, E_{i}^{max}\}$

- $\gamma=0.03$ annually; $E_{i}^{max}=0.60$ for radiology and workforce optimization and 0.80 for

workflow optimization.

- Bounded by clinical and operational constraints.

4. Economic Factors:
 - Healthcare inflation: UK 4.0%, Australia 5.0% (The King’s Fund and Australian

Government, 2022 projections).

*Source*: [The Health And Care Outlook For 2025 | The King’s Fund](https://www.kingsfund.org.uk/insight-and-analysis/blogs/health-care-outlook-2025)

[Health Funding Facts update | Australian Government Department of Health and Aged Care](https://www.health.gov.au/news/health-funding-facts-update)

- Currency volatility: ±5% annual standard deviation
 - GDP growth impact on healthcare spending: 1.2× GDP elasticity

5. Policy Scenarios:
 - Baseline: Current regulatory framework maintained
 - Accelerated: Government AI strategies fully implemented by 2027
 - Constrained: Increased regulatory oversight reduces adoption by 25%

**Annual projection calculations:**

$S_{ij}\left( h \right)=B_{j}\times w_{ij}\times\left( 1+\pi_{j} \right)^{h}\times A_{ij}\left( h \right)\times E_{ij}\left( h \right)\times\left( 1-R_{ij}\left( h \right) \right)$, $h=0,\ldots,6$.
where:
- $S_{ij}\left( h \right)$: projected savings in year $2024+h$

- $\pi_{j}$: country-specific annual inflation rate

- $A_{ij}\left( h \right)$: time-dependent adoption rate

- $R_{ij}\left( h \right)$: time-dependent implementation risk

- $E_{ij}\left( h \right)$: time-dependent effectiveness

**Uncertainty quantification:**
The above calculations were applied to each of the posterior samples produced by SMC, which results in a set of posterior samples for the annual projections. From these samples, the posterior means and credible intervals of the annual projections can be calculated.

**Key assumptions for 2024-2030:**
 - No major technological disruptions (e.g., AGI, quantum computing)
 - Political stability maintains current healthcare funding levels

- Regulatory frameworks evolve gradually without major reversals
 - International cooperation continues for AI standards development
 - Cybersecurity incidents do not significantly impact adoption

**Limitations:**
- Projections beyond 2028 have increased uncertainty due to technology evolution
- Currency projections assume current economic relationships persist
- Healthcare system structural changes not explicitly modeled
- Potential for non-linear adoption patterns in response to external shocks

**Table S2: Scenario impact summary**

| Scenario | UK cumulative savings (2024-2030) | AUS cumulative savings (2024-2030) | Key risk factors | Policy implications |
| --- | --- | --- | --- | --- |
| Baseline | $10.1B [9.1-11.1] | $8.0B [7.1-8.9] | Moderate implementation risks | Steady investment in AI infrastructure |
| High inflation | $9.1B (-9%) [8.2-10.0] | $7.3B (-9%) [6.5-8.1] | Healthcare cost escalation | Focus on cost-control measures |
| Low inflation | $10.7B (+6%) [9.7-11.8] | $8.4B (+5%) [7.4-9.3] | Economic stability | Accelerated AI deployment feasible |
| Currency volatility | $8.1B (-20%) [7.3-8.8] | $6.4B (-21%) [5.6-7.1] | Exchange rate fluctuations | Currency hedging strategies needed |
| Tech acceleration | $18.4B (+82%) [16.6-20.3] | $14.7B (+84%) [13.0-16.4] | Technology adoption risks | Aggressive R&D investment required |
| Regulatory constraint | $4.5B (-56%) [4.1-4.9] | $3.5B (-57%) [3.1-3.9] | Compliance costs, delays | Regulatory reform and harmonization |

**Table S3: Sigma Prior Sensitivity Analysis**

| Country | Sector | Sigma | Mean | Lower 95% CrI | Upper 95% CrI | % Change from σ = 0.15 |
| --- | --- | --- | --- | --- | --- | --- |
| UK | Radiology | 0.1 | 144.7 | 120.0 | 166.8 | 0.6 |
| UK | Radiology | 0.15 | 143.9 | 111.4 | 169.3 | 0 |
| UK | Radiology | 0.2 | 143.7 | 102.5 | 174.9 | -0.1 |
| UK | Workflow Optimization | 0.1 | 211.0 | 154.9 | 274.9 | -1.0 |
| UK | Workflow Optimization | 0.15 | 213.1 | 133.1 | 300.6 | 0 |
| UK | Workflow Optimization | 0.2 | 212.6 | 114.9 | 311.7 | -0.2 |
| UK | Workforce Optimization | 0.1 | 585.1 | 404.1 | 778.3 | -1.1 |
| UK | Workforce Optimization | 0.15 | 591.9 | 398.8 | 854.8 | 0 |
| UK | Workforce Optimization | 0.2 | 604.0 | 316.4 | 955.1 | 2.0 |
| AUS | Radiology | 0.1 | 80.1 | 68.7 | 92.7 | -0.4 |
| AUS | Radiology | 0.15 | 80.4 | 65.3 | 94.9 | 0 |
| AUS | Radiology | 0.2 | 80.2 | 62.0 | 98.0 | -0.3 |
| AUS | Workflow Optimization | 0.1 | 94.3 | 67.3 | 122.0 | -1.0 |
| AUS | Workflow Optimization | 0.15 | 95.2 | 62.4 | 135.8 | 0 |
| AUS | Workflow Optimization | 0.2 | 98.0 | 58.2 | 163.5 | 2.9 |
| AUS | Workforce Optimization | 0.1 | 558.1 | 401.9 | 719.3 | -0.6 |
| AUS | Workforce Optimization | 0.15 | 561.4 | 331.9 | 815.3 | 0 |
| AUS | Workforce Optimization | 0.2 | 565.5 | 309.7 | 838.7 | 0.7 |

**Table S4: Parameter Prior Sensitivity Analysis**

| Country | Sector | Perturbation | Mean | Lower 95**%** CrI | Upper 95**%** CrI | % Change from Reference |
| --- | --- | --- | --- | --- | --- | --- |
| UK | Radiology | Reference | 143.9 | 111.4 | 169.3 | 0 |
| UK | Radiology | A-20% | 143.9 | 110.0 | 168.3 | 0.0 |
| UK | Radiology | A+20% | 145.1 | 116.0 | 170.5 | 0.8 |
| UK | Radiology | R-20% | 144.7 | 113.9 | 172.2 | 0.6 |
| UK | Radiology | R+20% | 143.3 | 106.1 | 171.1 | -0.4 |
| UK | Radiology | E-20% | 143.9 | 111.2 | 170.1 | 0.0 |
| UK | Radiology | E+20% | 144.3 | 109.7 | 174.9 | 0.3 |
| UK | Workflow Optimization | Reference | 213.1 | 133.1 | 300.6 | 0 |
| UK | Workflow Optimization | A-20% | 209.3 | 119.4 | 305.3 | -1.8 |
| UK | Workflow Optimization | A+20% | 215.2 | 138.6 | 307.1 | 1.0 |
| UK | Workflow Optimization | R-20% | 209.8 | 121.3 | 301.6 | -1.5 |
| UK | Workflow Optimization | R+20% | 208.5 | 114.5 | 301.8 | -2.1 |
| UK | Workflow Optimization | E-20% | 210.5 | 126.9 | 299.4 | -1.2 |
| UK | Workflow Optimization | E+20% | 211.1 | 134.5 | 301.3 | -0.9 |
| UK | Workforce Optimization | Reference | 591.9 | 398.8 | 854.8 | 0 |
| UK | Workforce Optimization | A-20% | 592.5 | 384.9 | 882.7 | 0.1 |
| UK | Workforce Optimization | A+20% | 588.9 | 351.7 | 827.7 | -0.5 |
| UK | Workforce Optimization | R-20% | 593.4 | 369.5 | 847.3 | 0.3 |
| UK | Workforce Optimization | R+20% | 596.0 | 381.3 | 881.9 | 0.7 |
| UK | Workforce Optimization | E-20% | 594.9 | 380.3 | 889.4 | 0.5 |
| UK | Workforce Optimization | E+20% | 595.5 | 375.2 | 931.9 | 0.6 |
| AUS | Radiology | Reference | 80.4 | 65.3 | 94.9 | 0 |
| AUS | Radiology | A-20% | 80.7 | 65.4 | 99.2 | 0.3 |
| AUS | Radiology | A+20% | 81.0 | 65.7 | 98.6 | 0.7 |
| AUS | Radiology | R-20% | 80.9 | 64.7 | 101.4 | 0.6 |
| AUS | Radiology | R+20% | 80.4 | 64.7 | 96.6 | 0.0 |
| AUS | Radiology | E-20% | 80.6 | 61.9 | 97.1 | 0.2 |
| AUS | Radiology | E+20% | 80.9 | 66.7 | 99.8 | 0.6 |
| AUS | Workflow Optimization | Reference | 95.2 | 62.4 | 135.8 | 0 |
| AUS | Workflow Optimization | A-20% | 94.1 | 57.2 | 134.7 | -1.2 |
| AUS | Workflow Optimization | A+20% | 96.1 | 63.9 | 138.2 | 0.9 |
| AUS | Workflow Optimization | R-20% | 96.3 | 66.6 | 141.4 | 1.1 |
| AUS | Workflow Optimization | R+20% | 96.2 | 61.8 | 139.2 | 1.0 |
| AUS | Workflow Optimization | E-20% | 95.5 | 61.7 | 134.9 | 0.3 |
| AUS | Workflow Optimization | E+20% | 95.8 | 55.7 | 136.0 | 0.6 |
| AUS | Workforce Optimization | Reference | 561.4 | 331.9 | 815.3 | 0 |
| AUS | Workforce Optimization | A-20% | 556.6 | 347.4 | 787.2 | -0.9 |
| AUS | Workforce Optimization | A+20% | 571.7 | 394.7 | 787.8 | 1.8 |
| AUS | Workforce Optimization | R-20% | 562.7 | 344.3 | 827.5 | 0.2 |
| AUS | Workforce Optimization | R+20% | 565.3 | 359.8 | 810.4 | 0.7 |
| AUS | Workforce Optimization | E-20% | 564.6 | 320.9 | 851.6 | 0.6 |
| AUS | Workforce Optimization | E+20% | 561.1 | 335.3 | 814.7 | -0.1 |

**Note.** All values in $US millions. The last column measures percentage change from the posterior means reported in Table 1. Perturbation runs use *rng* seed = 43 (MATLAB twister) for reproducibility. SMC parameters: N = 1,000 particles, ESS threshold = 500, step-size = 0.01. Sigma prior held fixed at 0.15 × $y_{obs}$ for all perturbation runs.
