# Supplementary material for: Bayesian Analysis of AI-Driven Cost Savings in UK and Australian Health Care Systems: Cross-Sector Implementation Study
Source: JMIR Med Inform. 2026 Aug 26;14:e89113. doi: 10.2196/89113 (PMC13559155; doi:10.2196/89113)
Supplement: Supplementary file 4 [file medinform_v14i1e89113_app4.docx]

**Multimedia Appendix 4: Individual Parameter Distribution**

**Prior vs. Posterior Distribution Evolution Plots**
